# What we know about the actual implementation process of public physical activity policies: results from a scoping review

**DOI:** 10.1093/eurpub/ckac089

**Published:** 2022-11-29

**Authors:** Sarah Forberger, Lucia A Reisch, Biljana Meshkovska, Karolina Lobczowska, Daniel A Scheller, Janine Wendt, Lara Christianson, Jennifer Frense, Jürgen M Steinacker, Catherine B Woods, Aleksandra Luszczynska, Hajo Zeeb

**Affiliations:** Leibniz Institute for Prevention Research and Epidemiology—BIPS, Bremen, Germany; Leibniz Institute for Prevention Research and Epidemiology—BIPS, Bremen, Germany; Behavioural Economics and Policy, University of Cambridge, Cambridge, UK; Institute of Basic Medical Sciences, University of Oslo, Oslo, Norway; Domus Medica, Oslo, Norway; Department of Psychology in Wroclaw, SWPS University of Social Sciences and Humanities, Wroclaw, Poland; Division of Sports and Rehabilitation Medicine, Department of Internal Medicine, Ulm University Medical Center, Ulm, Germany; Division of Sports and Rehabilitation Medicine, Department of Internal Medicine, Ulm University Medical Center, Ulm, Germany; Leibniz Institute for Prevention Research and Epidemiology—BIPS, Bremen, Germany; Leibniz Institute for Prevention Research and Epidemiology—BIPS, Bremen, Germany; Division of Sports and Rehabilitation Medicine, Department of Internal Medicine, Ulm University Medical Center, Ulm, Germany; School of Health and Human Performance, Dublin City University, Dublin, Ireland; Department of Physical Education and Sport Sciences, Physical Activity for Health Research Cluster, Health Research Institute, University of Limerick, Limerick, Ireland; Department of Psychology in Wroclaw, SWPS University of Social Sciences and Humanities, Wroclaw, Poland; Melbourne Centre for Behavior Change, Melbourne School of Psychological Sciences, University of Melbourne, Melbourne, VIC, Australia; Leibniz Institute for Prevention Research and Epidemiology—BIPS, Bremen, Germany; Health Sciences Bremen, University of Bremen, Bremen, Germany

## Abstract

**Background:**

Physical inactivity rates have remained high worldwide since 2001. Public policies are an essential upstream lever to target individual physical activity (PA) behaviour. However, implementers have different strategies and face implementation challenges that are poorly understood. The present study analyzes the implementation processes of public policies to promote PA in terms of: (i) the policies covered and their legal quality, (ii) the actors and stakeholders involved in the implementation process and (iii) the used implementation strategies (vertical, horizontal or a mix).

**Methods:**

A scoping review was systematically conducted (registered Open Science Framework: osf.io/7w84q/), searching 10 databases and grey literature until March 2022. Of the 7741 titles and abstracts identified initially, 10 studies were included.

**Results:**

The current evidence includes high-income countries (USA, *n* = 7; UK, New Zealand and Oman, *n* = 1 each). Policy areas covered are education (school sector) and PA promotion in general (national PA plans or city-wide approaches). The legal classification ranges from laws (school sector) to coordination and budgeting to non-legally binding recommendations. The jurisdictions covered were federal (*n* = 4), state (*n* = 1), county (*n* = 1), school district (*n* = 1) and city (*n* = 3). Implementation strategies for city-wide approaches are characterized by a coordinated approach with vertical and horizontal integration; federal PA policies by a mix of implementation strategies; and the school sector by a strict horizontal top-down integration without the involvement of other actors.

**Conclusion:**

Implementation strategies differ by policy field. Therefore, continuous evaluation of the implementation process is necessary to align policy implementation with policy goals to promote individual PA behaviour.

## Introduction

Physical inactivity poses a significant risk factor for chronic diseases, comorbidity and premature death.^1^ In total, 7.2% and 7.6% of all-cause and cardiovascular disease deaths globally are attributable to physical inactivity.^2^ However, despite various behavioural approaches, physical inactivity has remained unchanged at a low level since 2001 worldwide.^3^^,^^4^

We note that a policy is an essential upstream determinant of individual health behaviours following Sallis et al.’s ecological approach to creating active communities. It is often necessary to address societal and complex problems.^5^ Policy instruments, such as information campaigns, recommendations, financial incentives or bans, are used by governments to directly or indirectly influence individual behaviour. Furthermore, by actively curating the built environment based on behavioural insights,^6^^,^^7^ governments can create a physical activity (PA)-friendly and -promoting environment, encouraging individuals to adopt a more physically active lifestyle.^6^

**Figure 1:**
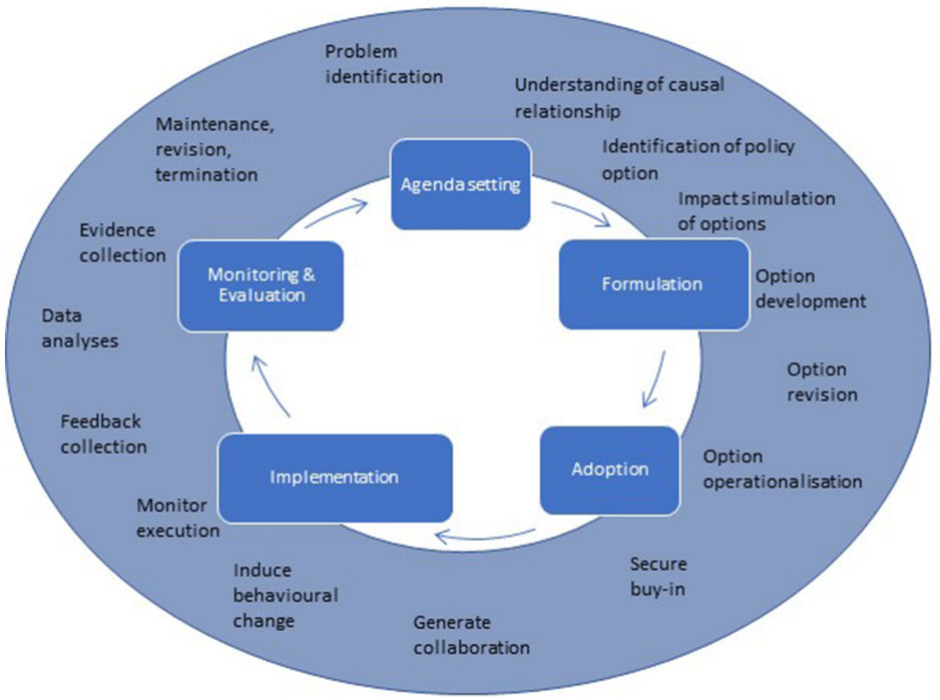
Policy cycle heuristic and typical accompanying activities (own figure adapted from Armenia et al. (2014))^13^^,^^14^

To be effectively implemented, policies need more than problem formulation, agenda-setting and adoption of a respective regulation (figure 1 and Supplementary appendix S4). One key is a well-designed implementation process. Policy implementation is generally defined as a series of activities carried out by the government, its agencies or institutions mandated or subordinated to achieve the goals formulated in the policy statements.^7^ Here, we define policies as purposeful decisions, plans and actions made by voluntary or authoritative actors in a system designed to create system-level changes to directly or indirectly achieve specific societal goals. Within this definition, *public policy* is a form of government action usually expressed in e.g. a law, a regulation, a guideline or a recommendation that reflects the government’s intent or its representative entities.^8^ Given their regulative power, public policies can be issued by federal, state or regional (e.g. county, city level) authorities. The policies can be placed on a continuum between the minimum degree of coercion, such as guidelines and recommendations (soft), to a maximum degree of coercion, like laws (hard), with coercion measuring ‘the extent to which a tool restricts individual or group behaviour as opposed to merely encouraging or discouraging it’.^9^ Different levels of coercion and the associated perception of coercion lead to varying degrees of (perceived) intrusion into the individual’s life. Choice architecture interventions that subtly change the environment in which individuals make decisions can reduce the perception of intrusion.^10^^,^^11^ An example is the Active Design approach to neighbourhoods, streets and outdoor spaces that encourage walking, bicycling and active transportation and recreation to increase PA (https://www1.nyc.gov/assets/planning/download/pdf/plans-studies/active-design-guidelines/adguidelines.pdf; access: 26 July 2022). Also, incentives or poster prompts to use stairs are part of this category.^12^

Using the policy cycle as a heuristic (figure 1), we see that the implementation phase lies between the stages of problem definition, agenda-setting and policy formulation and the later stages of evaluating the impact and effectiveness of the policy, then maintaining the policy or terminating it. It is about who does what, when, how and why; actions lead to responses and consequences, bridge the gap between policy action and impact, and establish a link between policy intent and outcome.^15^ Most political decisions are based on the implicit assumption that implementation will occur. However, the case of symbolic politics can be encountered as well.^16^ In this case, the policy takes on a symbolic character for the public, and no implementation is sought.^17^

**Figure 2:**
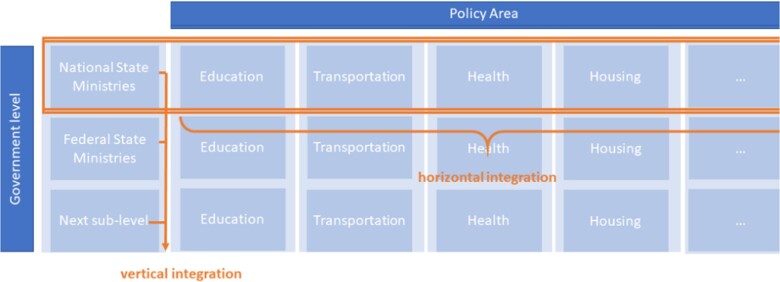
Horizontal and vertical integration (own figure, adapted from Kettner et al. (2012))^41^

Implementation can take place within and across government levels (jurisdiction). Horizontal integration is defined as the mainstreaming of core elements of the policy into other policy areas on one level of the government (e.g. between different ministries). Vertical integration is defined as incorporating core policy elements within one policy area into the next level of government and administration. Vertical policy integration provides a platform linking bottom-up and top-down dynamics (table 1 and figure 2).^18–20^

**Table 1:** Implementation strategies for vertical integration

	Focus	Characteristics
Top-down	The main emphasis is on the ability of decision-makers to produce unequivocal policy objectives and on controlling the implementation stage	Implementation style: centralized Executed by hierarchically ordered administrative structures Less leeway and little deviation Hierarchical guidance Driven by elitist
Bottom-up	Local bureaucrats as the main actors in policy delivery and conceive of implementation as negotiation processes within networks of implementers	Implementation style: decentralized Leeway for street-level bureaucrats (implementers) to adapt to local contexts and circumstances Problem-solving approach Participatory approach
Hybrid	Incorporating elements of top-down, bottom-up and other theoretical models	Elements of both approaches Including external factors (e.g. advocacy coalition framework) Implementation process not viewed in isolation and considering exogenous influences

The process of policy implementation itself is dynamic and non-linear, with feedback loops that enable policy learning and encompass elements of all previous stages of the policy process, including all associated uncertainties and contingencies.^21^

Successful implementation depends on various factors, such as public institutions’ commitment, capacity and resources.^22^ Based on the literature, policies, directly and indirectly, affect the PA of the population.^23^ However, the actual impact of policies depends on their implementation. To the best of our knowledge, no review has analyzed the implementation processes of public policies on PA promotion. The work presented in this paper fills this gap with an analysis across different policy areas in terms of (i) PA policies covered and their legal quality; (ii) actors and stakeholders involved in the implementation process; and (iii) implementation strategies (vertical vs horizontal). To date, very little empirical evidence has been compiled on how such implementation processes work. Typically, they are not evaluated, or the results are not published. However, it is of significant interest to policymakers to know about barriers to and drivers of success. The present paper aims to review available evidence from implementation cases of PA policies and derive some guiding notes on how to best implement policies promoting PA.

## Methods

The scoping review follows an approach similar to a systematic review. To depict the flow of information through the different phases of the scoping review, we used the Preferred Reporting Items for Systematic Reviews and Meta-Analysis: Extension for Scoping Reviews (PRISMA-SCR and flowchart).^24^ A study protocol was published beforehand with Open Science Framework.^25^

### Search strategy and information sources

An experienced information specialist in the review team developed the search strategy and searched. Appropriate keywords and synonyms and controlled vocabulary were combined to create a structured search covering the concepts of PA promotion, public policy and the implementation process. The search syntax and controlled vocabulary were adapted for searches in other databases on other platforms. No limits in publication date or study design were applied. No language restrictions were used during the search and title/abstract screening as all papers indexed in the databases provided an English abstract. Papers not published in English, German, French, Norwegian or Polish were excluded during the full-text screening due to missing language skills in the reseach team and marked accordingly. The searches were conducted in the following electronic databases during February 2020 and updated until 25th March 2022: Medline, EMBASE, PsycInfo, CINAHL, EconLIT, ASSIA, ERIC, PAIS, SSCI, SCI-Expanded, A&HCI, BKCI-S and BKCI-SSH. The search strategy for Medline and a detailed overview of the number of hits per databases are available as a Supplementary Appendix S1. In addition to traditional bibliographic databases, three grey literature sources were included in the results (OpenGrey, ThinkTank, BASE and Google Scholar) to reduce publication bias and increase comprehensiveness. Due to limited resources, the grey literature research was not updated. The search syntax was adapted for each website. The references of included studies and previously published reviews and studies were hand-searched for additional citations. All references were integrated into the PRISMA flow chart. All results were exported to EndNote reference management software for de-duplication. De-duplicated results were imported to Covidence systematic review management software for title/abstract and full-text screening. All screening was conducted independently by two expert reviewers. Conflicts were resolved by a third person not involved in screening the paper in question. If the conflict could not be resolved, a third person not involved in the screening process was available for discussion.

### Inclusion and exclusion criteria

We included all papers available in full text that report on the implementation process of PA policies issued by governments or governmental agencies or subordinated or subcontracted institutions independently of the sector/setting. No language or study-type restrictions were applied. If an article had to be excluded due to a lack of language proficiency in the review team, it was marked accordingly. Papers reporting on intervention implementation processes that were not based on public policies were excluded due to our focus on the public policy implementation process.

### Data extraction, coding and analyses

Collected data were categorized into the following groups: developmental policy background (pre-existing policies, pre-existing collaborations among stakeholders), instruments used in the implementation process, the course of the implementation process and reported outcomes (short, medium and long term). Detailed information and the extraction sheet were pre-registered in the protocol and can be found with theoretical reasoning and references in Supplementary Appendix S2, Table SA2. A narrative synthesis of the included studies was used to analyze and interpret the data.

## Results

A total of 15 462 publications were identified, with 7721 excluded as duplicates. The remaining 7741 titles and abstracts were screened; 7540 were excluded, leaving 201 full texts for screening. One hundred ninety-one publications were excluded during the full-text screening, leaving 10 publications for synthesis (Supplementary appendix S3). The most common reasons for exclusion were: (i) the term ‘implementation process’ was mentioned, but the process itself was not described (*n* = 93); (ii) no public policy (*n* = 43); (iii) wrong outcome (policy in general, no focus on PA, nutrition only, Non-Communicable Diseases (NCDs) in general) (*n* = 30); (iv) language reasons (*n* = 14); and (v) no empirical research (letter to the editor, editorial, commentary, methods paper) (*n* = 10). Descriptive details of the included articles can be found in the Supplementary material.

### Geographic distribution, frameworks used and study designs

Of the included papers, seven studies were conducted in the USA,^22^^,^^26–31^ and one each in the UK, New Zealand and Oman.^32–34^ The jurisdictions included federal (*n* = 4),^26^^,^^29^^,^^33^^,^^34^ state (*n* = 1), county level (*n* = 1),^22^ school district (*n* = 1)^31^ and city level (*n* = 3).^28^^,^^30^^,^^32^

For the analyses, one study used Rist’s theoretical triad,^33^ one used the socio-ecological model as an analytical framework^27^ and another drew on the literature on policy implementation and planning.^22^ The study covering Oman used a combination of the PA content analysis grid, the health-enhancing PA policy audit tool and the policy cube approach for diet-related NCDs as guiding frameworks for their analysis.^34^ The other studies did not mention using any theory, model or framework as guidance.

All except one study used a case-study design. The exception is the overview report by Bozzo et al.,^26^ which summarized the development of fitness and PA policies in the USA up to 1981. Methods used in the studies (if reported) were formative process evaluation,^29^ document analysis,^34^ policy review,^22^ and a combination of interviews and document analysis.^33^

### Public policy characteristics

The included studies can be divided into two broad areas: PA promotion for the general population and PA promotion in schools.^27^^,^^31^ The first area can be further divided into two subgroups: available national approaches to PA promotion,^26^^,^^29^^,^^33^^,^^34^ such as the US National Physical Activity Plan (NPAP) or the National Physical Activity Plan in Oman; and public policies that focus on urban design^30^ or city-wide approaches^22^^,^^28^^,^^32^ (table 2).

**Table 2:** Study characteristics

Country	Level	Policies covered	Policy areas/actors involved	Policy instruments	Horizontal/vertical implementation
USA	National	All policies that promote fitness and PA until 1981	Various [health, other Federal Agencies (Interior, Heritage Conservation and Recreation Services, National Park Service, Land Management, Education, Agriculture, Housing); other independent Agencies]	Communicative, regulative, economic (minimum)	Horizontal and vertical, no pre-existing structures were reported
Bozzo et al.^26^					
USA	National	NPAP	Sport + private and community sector	Not mentioned	Vertical within sectors, existing structures reported
Evenson and Satinsky^29^					
New Zealand	National	Kiwi sport	Sport	Regulative, economic	Vertical, existing structures reported
Keat and Sam^15^					
Oman	National	National Physical Activity Plan	Starting with four government sectors (education, health, sports and municipalities at national and sub-national levels) sectoral involvement expanded to include transport, housing, higher education	Regulative (not legally binding)	Vertical and horizontal, using existing structures (part of the national NCD policy)
Al Siyabi et al.^34^					
USA, Mississippi, Tennessee	Federal state, school level	School bill	Education (schools)	Regulation (law)	Vertical, no pre-existing structures were reported
Dyson et al.^27^					
USA, Maryland,	County	Local policies to increase PA	Various (transportation, education, city planning, health, and recreation)	Regulation (coordination)	Horizontal and vertical, existing structures reported
Montgomery County					
Salvesen et al.^22^					
USA, Wyoming, Arizona, Minnesota, New Mexico, Texas	School districts	Local Wellness Policies	Education (schools)	Regulation (law)	Vertical
Pitt Barnes et al.^31^					
USA, Texas, San Antonio	City	NPAP (local adaption)	Various + private and community sector	Regulative (coordination), economic	Horizontal and vertical, existing structures reported
Esparza et al.^28^					
USA, New York	City	Active Design Guidelines	Various + private sector	Communicative, regulative (coordination)	Horizontal and vertical, existing structures reported
Lee^30^					
England, Liverpool	City	Liverpool Active City Strategy	Various + private and community sector	Communicative, regulative, economic	Horizontal and vertical, existing structures reported
Dawson et al.^32^					

The policies examined have different legal qualities. In the school sector, the two papers included reports on the implementation process of national laws.^27^^,^^31^

Kiwi Sports is described as a national programme in New Zealand underpinned by government regulation and budget.^33^ Implementing the National Physical Activity Plan in Oman is described as a national recommendation formally adopted by the government, but it is not legally binding.^34^

The NPAP implementation utilizes a public–private collaborative partnership involving private-sector organizations and government agencies (^35^; at the local level: Esparza et al.^28^; at the national level: Evenson and Satinsky^29^). According to the plan, ‘no single, central organisation will be responsible for implementing the plan or providing the necessary funding. Instead, Americans—as individuals or through their organisations or government agencies—will implement the plan's strategies and tactics in ways that benefit everyone’.^36^

Dawson et al.,^32^ Lee,^30^ Esparza et al.^28^ and Salvesen et al.^22^ reported that coordinated approaches at the local level are more akin to whole-of-city approaches and Active Design guideline implementation with strong public-sector involvement (without legal regulation in the form of legislation, but with administrative coordination as well as financial support).

Bozzo et al.^26^ document all types of regulation and policy types, as it gives an overview of the development process of fitness and PA policies in the USA until 1981.

### Pre-implementation system-level context and use of pre-existing system-level structures

In the school setting, legislation stemming from the Child Nutrition and WIC Reauthorization Act of 2004 was passed in Tennessee and Mississippi in 2006 and 2007. The laws were passed shortly before the mandatory federal implementation of the Wellness Guidelines for Nutrition and Physical Activity at the beginning of the 2006–07 school year.^27^^,^^31^

Pre-existing policy structures were reported for the NPAP, Kiwi Sports in New Zealand and Oman’s National Physical Activity Plan.^29^^,^^33^ The Oman National Physical Activity Plan is the only initiative also involved in the fight against non-communicable diseases.^34^

The authors described a long tradition of cross-sector collaboration for the whole-city approaches (San Antonio, New York, Liverpool) and a county-level plan in Maryland. The initiatives assessed are based on long-term relationships and partnerships.^22^^,^^28^^,^^30^^,^^32^

### Implementation strategies (horizontal/vertical integration) and actors involved

In four cases, only vertical integration (a top-down approach) was mentioned: two cases within the school sector,^27^^,^^31^ the implementation of the NPAP where the implementation process took place within but not between the specific sectors^29^ and the reorganization of Kiwi sports.^33^ No horizontal integration was reported for these four cases, i.e. no implementation and coordination between authorities at the same level of jurisdiction.

All other cases reported vertical and horizontal integration involving different administrative jurisdictions.

Seven of 10 cases involve different policy areas such as urban planning, architectural design, active transport, recreational facilities or land use.^22^^,^^26^^,^^28^^,^^29^^,^^32–34^ The NPAP at the national and local levels and city-level implementation approaches (support of Active Design guidelines, ‘Active City Liverpool’) contain the involvement of private and community sector representatives.

The studies reporting the implementation of the school bills in Tennessee and Mississippi^27^ and the implementation of the Local Wellness Policies in several US states,^31^ as well as the study covering the reorganization of Kiwi Sports,^33^ named no involvement of other policy fields and actors.

Regarding policy instruments used within the implementation process, legislation was explicitly mentioned in the two education-sector cases.^27^^,^^31^ In two other cases, regulatory means were coordinated without laws.^22^^,^^34^ In five cases, two or more instruments combined regulatory (coordination) and economic or information instruments, or all three categories of instruments.^1^^,^^22^^,^^26^^,^^32^^,^^33^ One study used regulatory tools without legal binding force.^34^ For one study, no instrument was mentioned.^29^

Implementation actors were administrative staff,^22^^,^^26^^,^^30^^,^^34^ sector members,^29^ other organizations,^32^^,^^33^ school staff^31^ and professional urban planners.^30^ At the city level, engaged in implementing the NPAP, the New York Active Design guidelines and the Liverpool Active City Strategy, the involvement of private and community members as implementation actors was reported.^28^^,^^30^^,^^32^

### Short, medium- and long-term outcomes reported for the policies

The published results varied. In the study of the Liverpool whole-city approach, behavioural data were reported. These were not significant but showed a positive trend towards more PA.^32^ Pitt Barnes et al.^31^ reported implementing local wellness interventions in six US school districts, observing that some implementation had occurred, but no school implemented the whole policy.^31^ The findings reported by other studies were implementation outcomes. The development of networks and friendships between stakeholders and actors as a positive outcome of the implementation process was reported.^1^^,^^28^ Keat and Sam^15^ documented a change in the system due to changes in funding structures. A complete failure of implementation occurred due to a reduction in the necessary budget for the school policies in the US states of Tennessee and Mississippi.^27^ No outcomes of the Oman plan were reported.^34^

## Discussion

This study aimed to analyze the implementation processes of public policies to promote PA in terms of (i) PA policies covered and their legal quality; (ii) actors and stakeholders of the implementation process; and (iii) implementation strategies (vertical vs horizontal integration, mix of the two).

The data presented are fragmented and do not follow unified standards. Moreover, only results from high-income countries are shown based on the studies included. Oman, however, is a Near East country located on the east side of the Arabian Peninsula that fundamentally differs from the other states in its political structures, cultural norms and weather conditions.^34^

While the implementation process evaluation is a highly active research field within the intervention and implementation research,^37^ the main focus is the evaluation of single or complex interventions with limited attention to public policies. The small number of studies we could include in our review mirrors this significant research gap. Knowledge in this area could improve our understanding of the role of political systems and state action within the complex system of variables influencing individual behaviour. This gap is increasingly recognized, and work has started to evaluate the implementation of public policies or use systemic approaches to PA analysis. Recently, Lobczowska et al.^38^ reviewed different applicable frameworks for policy implementation. However, the methodological approaches need to be further advanced in establishing comparability. Mixed methods and Big Data approaches are possible in the future and will impact efforts to standardize reporting of the evaluation process and results. Noticeably, early studies seldom use frameworks. This has changed over the years. For example, the work of Al Siyabi et al.^34^ is based on well-known and frequently used frameworks in the field of PA promotion; these frameworks significantly improve understanding and the comparability of results. Work that encompasses both implementation research and comparative public policy is currently underway within the Policy Evaluation Network PEN to further standardize how policy implementation evaluations can be conducted and reported in PA promotion (currently non-public deliverables). However, one of the most significant challenges is to increase comparability without negating the complexity in the individual cases.

### Actors and stakeholders involved in the implementation process and implementation strategies

In the cases in which urban planning and city approaches were reported,^22^^,^^28^^,^^30^^,^^32^ there seems to be an opportunity to integrate implementation strategies involving different policy areas, administration sectors, professional associations and civil society. Both horizontal and vertical integration is used. Although the health departments have taken over the coordination, they bring together representatives from administrative sectors such as transport, urban planning, sports and land use with representatives from civil society and professional associations. The focus on planning and, in Liverpool^32^ and San Antonio,^28^ on a holistic approach for the entire city might mitigate the normative focus on health and the associated conflicts, e.g. the resources required for implementation. PA was seen more as mobility behaviour and less as a health-promoting lifestyle.^22^ Also, all three city-level studies report an already long history of informal and formal structures that facilitated coordination.^28^^,^^30^^,^^32^

Coordination between different levels of government and agencies helped to achieve policy goals. For effective coordination, authorities must have a common goal and a desire to work together.^39^ Considering the obstacles within and between administrations, such as conflicting roles, lack of resources, personal differences and the lack of a spokesperson, it is remarkable how much coordination was reported in the studies. This was based on necessity because projects initiated by the administration could only be implemented in cooperation with different policy areas and were based on a long tradition of cooperative approaches.^22^

The picture is different for the school sector, where results show approaches at the federal and school district levels. At the federal level, a strong top-down approach prevails. The study in the US states of Mississippi and Tennessee notes that implementation is expected to be driven by the law’s obligation to implement the policy. The administrative mechanism takes off on its own. However, teachers’ feedback has shown that the lack of resources, time and, above all, the intense focus on children’s academic performance are obstacles to the implementation of PA policies.^27^

The study on the implementation processes at the school-district level described a partial implementation and reported variables in intervention research such as lack of coordination, time and resources to support implementation processes. It is also noted that no school has implemented all the targets.^31^

However, no or very little cooperation between different policy areas was reported in the two studies within the school sector. One can speculate that this is due to the very narrow objective of the policy, which focuses mainly on PA in schools. However, perhaps it also might be due to how education policy has been implemented for many years (path dependency). This replicates the results found by Woods et al.^23^ in her work about PA in schools.

It could be argued that the more significant the role of policy prioritization versus administrative implementation, the more difficult the actual implementation gets. In most cases, implementation is done (or begun) by an administration that is not responsible for the legislation.^40^^,^^41^ Therefore, it is essential to consider both the political dimension and administrative implementation. The area of implementation processes must not be neglected compared with politically desired goals. In that case, there is a risk of creating a gap between politically anticipated goals and implementation capacity by the administration.

### Limitation

Due to the limited number of studies on this topic, it is impossible to generalize the results to policy-field-dependent patterns for implementation processes. Yet the different processes in the school sector and the Active City approaches are striking. We may have missed some studies due to the limitations of our search strategy.

## Conclusion and future research

Abstracting these results could lead to different approaches in various policy fields in the coming years. For example, structures must first be created for cooperative strategies in the school sector. In the area of whole-city approaches, which are characterized by vertical and horizontal integration and include many actors, the aim would rather be to stabilize the cooperation. This can be done either as political action in the framework of law or as a fixed component of public action with an allocated budget to strengthen the approaches and establish them over the long term. Another major goal is to reduce the stress on the actors and the uncertainties due to the lack of funding. Health in All Policies approaches would be well suited for this.

In the case of national action plans, such as in Oman, it remains to be seen whether public–private partnerships will develop, as in the USA, to link social and governmental actors and to support the plans and their implementation and enforcement. Without legal enforcement power, the Oman plan’s basic application may be challenging. However, with choosing to link the national plan to the non-communicable disease initiative, other plans or legislation to increase the pressure for implementation without having to create binding regulations can be used. Time will tell, and continuous evaluation of the developmental processes is necessary. Parallel to the dynamic evolution of implementation strategies and legal frameworks, analytical methods will adapt and give more space to mixed methods, and perhaps Big Data approaches in the future to be used in PA implementation evaluation.

## Supplementary data

Supplementary data are available at *EURPUB* online.

## Supplementary Material

ckac089_Supplementary_Data
