# Supplementary material for: What we know about the actual implementation process of public physical activity policies: results from a scoping review
Source: Eur J Public Health. 2022 Nov 29;32(Suppl 4):iv59–65. doi: 10.1093/eurpub/ckac089 (PMC9706118; doi:10.1093/eurpub/ckac089)
Supplement: ckac089_Supplementary_Data [file ckac089_supplementary_data.zip › ckac089_Supplementary_Data/Forberger_PAmap_Appendix1_Query.docx]

## Appendix 1

**Database Query, Number of hits per Database** **and Database details**

MEDLINE(R) and Epub Ahead of Print, In-Process & Other Non-Indexed Citations, Daily and Versions(R) 1946 to February 5, 2020 via Ovid
Searched/exported 06.02.2020 (updated 25.03.2022)

| **Search line** | **Query** | **Results 2020** |
| --- | --- | --- |
|  |  |  |
| 1 | ("public polic*" or law* or "action program*" or regulation* or guideline* or "action plan*").ti,ab. | 1.309.626 |
| 2 | ((nation* or state* or government*) adj5 (policy or policies or policymak* or "policy mak*" or strateg* or recommendation*)).ti,ab. | 45.282 |
| 3 | exp public policy/ | 137.412 |
| 4 | exp policy making/ | 25.295 |
| 5 | or/1-4 | 1.468.739 |
| 6 | (implement* or enforc* or adapt* or application* or apply or applies or "policy formulation*" or "policy formation*" or "policy change*" or scale-up).ti,ab. | 2.152.253 |
| 7 | 5 and 6 | 193.033 |
| 8 | (sport* or exercis* or "physical fitness" or "physical activit*").ti,ab. | 425.761 |
| 9 | exp sports/ | 178.623 |
| 10 | exp "physical education and training"/ | 13.359 |
| 11 | exp exercise/ | 188.824 |
| 12 | or/8-11 | 550.699 |
| 13 | (prevention* or promotion* or initiative* or intervention* or campaign*).ti,ab. | 1.550.791 |
| 14 | exp "health promotion"/ | 75.118 |
| 15 | 13 or 14 | 1.584.253 |
| 16 | 12 and 15 | 108.472 |
| 17 | 7 and 16 | 2.830 |

**Number of hits per Database**

| **Database** | **Original search, number of hits** | **Deduplicated search, number of hits** |  |
| --- | --- | --- | --- |
|  |  |  |  |
| Medline | 2.830 | 2.815 |  |
| EMBASE | 1.896 | 664 |  |
| PsycInfo | 946 | 437 |  |
| CINAHL | 1.452 | 275 |  |
| EconLit | 65 | 33 |  |
| ASSIA | 507 | 232 |  |
| ERIC | 265 | 157 |  |
| PAIS | 217 | 95 |  |
| WoS | 2.014 | 82 |  |
| Scopus | 1.513 | 1.016 |  |
|  |  |  |  |
|  | **11.705** | **5.806** | **total** |
|  |  | 5.899 | discards |

**Database details**

| **Database** | | **Provider** | **Timespan – until 25.03.2022** |
| --- | --- | --- | --- |
| **1** | Medline | Ovid | 1946- |
| **2** | EMBASE | OVID | 1947- |
| **3** | PsycInfo | Ovid | 1806- |
| **3** | SSCI & SCI-Expanded | Web of Science | 1900- |
|  | A & HCI |  | 1975- |
|  | BKCI-S |  | 2013- |
|  | BKCI-SSH |  | 2013- |
| **4** | CINAHL | Ebsco | 1981- |
| **5** | EconLIT | Ebsco | 1886- |
| **6** | ASSIA | ProQuest | 1987- |
| **7** | ERIC | ProQuest | 1966- |
| **8** | PAIS | ProQuest | 1914- |
| **9** | SCOPUS |  | 1970- |
