# Supplementary material for: What we know about the actual implementation process of public physical activity policies: results from a scoping review
Source: Eur J Public Health. 2022 Nov 29;32(Suppl 4):iv59–65. doi: 10.1093/eurpub/ckac089 (PMC9706118; doi:10.1093/eurpub/ckac089)
Supplement: ckac089_Supplementary_Data [file ckac089_supplementary_data.zip › ckac089_Supplementary_Data/Forberger_PAmap_Appendix3_Flow Chart.docx]

# Appendix 3

Records excluded
(n = 191)

Implementation mentioned but no implementation process described (n=93)

No public policy (n=43)

Wrong outcomes (n=30)

Language (n=14)

Erratum/Commentary/Editorial Study Protocol/Conference Paper (n=10)

No pdf (n=1)

**Flow diagram**

Records identified through database search
(n =15,462)

Records excluded
(n = 7,540)

Additional records identified through other sources
(n =230)

Studies included
(n = 10)

Full-text articles assessed for eligibility
(n = 201)

Title/abstract screening
(n = 7,741)

Records after duplicate removal
(n =7,741)

## Identification

## Eligibility

## Included

## Screening
