# Supplementary material for: What we know about the actual implementation process of public physical activity policies: results from a scoping review
Source: Eur J Public Health. 2022 Nov 29;32(Suppl 4):iv59–65. doi: 10.1093/eurpub/ckac089 (PMC9706118; doi:10.1093/eurpub/ckac089)
Supplement: ckac089_Supplementary_Data [file ckac089_supplementary_data.zip › ckac089_Supplementary_Data/Forberger_PAmap_SuplementalMaterial_studie_characteristics.docx]

### Supplement

Table S1: Detailed study characteristics

| **No.**  **Author**  **Origin/ Jurisdiction**  **/Legal quality** | **Name of the policy**  **Content** | **Study design**  **Framework used**  **Data used** | **Policy area/actors involved** | **Pre-existing structures**  **Implementation process** | **Target group** | **Policy instrument**  **horizontal vs Vertical integration** | **Outcome reported** |
| --- | --- | --- | --- | --- | --- | --- | --- |
| 1  Bozzo et al. 1981  USA/national/all | all policies that promote fitness and PA until 1980  all policies that promote fitness and PA until 1980 | case studies  non-reported  Document analysis | Health and other Federal Agencies (Interior, Heritage Conservation and Recreation Services, National Park Service, Land Management, Education, Agriculture, Housing) and independent Agencies | No  - system characterised by diffusion in the responsibilities of Federal agencies  - fragmented and short-lived with a different system, strategies and philosophies  - the absence of an overarching policy decision-maker and implementer  - states do seem to follow their own chosen course and areas of emphasis  - focus traditionally on the school setting | administrative staff | communicative, regulative, economic  horizontal & vertical | Non-reported |
| 2  Evenson et al., 2014  USA/ National/ set of policies, programs, and initiatives | US National Physical Activity Plan (NPAP)  A comprehensive set of policies, programs, and initiatives  - 5 overarching strategies and 44 specific strategies with corresponding tactics | case study (formative process evaluation)  non-reported  reports, in-depth interviews (n=12) | Sports, private and community sector | Yes  - six months after the NPAP launch, National Coalition for Promoting Physical Activity (NCPPA) released a companion implementation plan called “Make the Move” -> identified measurable outcomes and objectives for each of the sectors ->some (not all strategies) 1-year and 5-year goals, and tactics to achieve them  - sector working lists form the national conference as a starting point to populate the sector teams with regular meetings  - sector Mass Media and Volunteer/NGOs -> no implementation plan  - sectors structure implementation and organisation by their own  - Leadership changes due to time constraints  - different communication strategies within sectors  - hardly any cross-sector collaboration  - no NPAP funding in 2011 but in-kind work from individuals and organisations | sector members | Non-reported  vertical within sectors | - discussion within the health promotion community around other issues apart from NPAP goals - development of a sense of camaraderie among people involved - network building |
| 3  Keat et al., 2013  New Zealand/national/ Government funding initiative | Kiwi sport  initiative to get more school-age children involved in organised sport | case study (interviews and document analyses)  Rist’s (1998) theoretical triad  Semi-structured interviews | school, local | Yes  - government-> trigger SPARC to revise its funding strategy - funding is now distributed as a bulk sum to each RST  - RST is still accountable to SPARC, the new investment strategy and funding arrangement grant RSTs more autonomy and more discretion in where money is allocated within their communities  -> RSTs are to undergo a thorough consultation process with their communities to determine regional needs and wants and produce a plan to present to SPARC for approval before funds are released.  - all funds delivered through the RPF to RSTs are to be redistributed into the community; no money for administration | RST | regulative, economic (funding)  vertical | - use of funding to change the system -> essential function of the RSTs has been to shape the policy objectives coming from the government into their regional mould (torn between community needs and government goals) - the problem of unsustainability -> lack of accessible money in the community, and frequently changing nature of the sports sector (especially surrounding pa and mass sport policy)-> short funding tenure contributes to the reduction in the ability of trusts to plan with any forward certainty and affects the confidence of other funders in the trust’s long-term sustainability |
| 4  Al Siyabi et al. 2021  Oman/national/plan | National Physical Activity Plan  to reduce physical inactivity in adults by 10% and provides a clear timeframe (2016 – 2025) | case study, document analysis  physical activity content analysis grid, the health-enhancing physical activity policy audit tool, and the policy cube approach for diet-related NCDs  National NCD policy, the physical activity plan of action, and two WHO Mission Reports |  | Yes (part of the NCD programme)  - early implementation - national sub-committee on physical activity was fully established, overseen by the National NCD committee  - sectoral involvement expanded to include transport, housing, higher education  - but missing budget for implementation | administrative | Regulative (governmental approved but not legally binding)  Vertical and horizontal | Non-reported |
| 5  Dyson et al., 2011  USA, Mississippi, Tennessee/ federal state, school level/law | Tennessee, House Bill 3750 and Senate Bill 3991  to engage K-12 students in 90 minutes of PA during the school week | case study  social-ecological model  interviews (n=73), direct observation of PE classes and other school-based activities, field notes, PE audit, document analyses | schools | No  For both states:  State-level:  - general assumption to comply, despite lack of goals, accountability, and funding -> to enact the mandate  School-level:  Administration  - no priority -> no funding  - no direction on how to enact new policy  - no accountability measures (evaluation, reporting mechanism)  no uniform understanding of the policy (varying level of awareness and understanding, support, attempts to comply)  - academic pressure to fulfil NCLD requirements (do have accountability standards) -> no room for anything else  Teacher level:  - marginalised status of PE  - missing, few resources  - narrow curriculum (callisthenics, basketball, weight training, running) -> increase marginalisation of kids, no money for equipment  Student level:  - marginalisation of kids, that is not good -> narrow curriculum  - PE is not seen as an actual class |  | Regulative  Vertical | -no specific goals or objectives and no funding or compliance mechanisms ->. Observed implementation failure in all eight schools |
|  | Mississippi Public Schools Standard 32, mandated in 2004  made PE a required course for high school students |  | schools |  |  | Regulative  Vertical |  |
| 6  Salvesen et al., 2008  USA, Maryland, Montgomery County/ County (local)/local policies | Local policies  Increase PA | case study (policy review)  literature on policy and planning implementation  interviews (with 26 individuals) | transportation, education, city planning, health, and recreation | Yes  - Informally established operating procedures and guidelines on a variety of tasks and activities (from land development approval to sidewalk snow clean-up responsibilities and the removal of trash bins from public parks)  - Guidelines for new development  - long-term process  - Development of a variety of mechanisms to facilitate greater coordination across government agencies, between the county and local governments, and between agencies and residents  - county coordinates-> opportunity for each agency to comment on the project and to angle for additions or modifications that help them achieve their mission -> mandatory referral process | planning staff/administrative staff | regulative (coordination)  horizontal & vertical | Non-reported |
| 7  Pitt Barnes et al., 2011  USA 2 = Wyoming, 1 each Arizona, Minnesota, New Mexico, Texas/school districts/law | Local Wellness Policies (LWP)  nutrition education, physical activity, and other school-based activities designed to promote student wellness | case study  non-reported  interviews in 6 districts  (n= 88) and 12-site visit reports | schools | No  - All six districts limited opportunities for physical activity across all grade levels, although most elementary schools in all districts allocated 20 to 40 minutes  - most schools were complying  - No LWPs addressed physical activity opportunities for students in afterschool programs | school staff | Regulative  vertical | - 6 LWPs were consistent with the federal mandate, although they varied in content and degree of specificity - none fully implemented - all six districts offered students only limited opportunities for physical activity, and all six collected data to monitor the process and outcomes of their LWPs |
| 8  Esparza et al., 2014  USA, Texas, San Antonio/city/programme and initiatives | San Antonio adaption of the US National Physical Activity Plan (NPAP)  - 3- to the 5-year master plan  - a roadmap for transforming the San Antonio area into an active living community | case study  non-reported  not reported | local health department, further departments, private and community members | Yes  - discussion about sustainability beyond grant deliverables  - ALCSA: continue as a volunteer organisation after separating from LHD -> strategic planning sessions to develop short- and long-term goals to support ALCSA’s mission and vision and advance plan implementation  - use of meaningful developed relationships throughout the project, promoting its integration in the community and sustainability  - ALCSA integrated into local agencies, including MFC, and the plan’s strategies were incorporated into LHD initiatives  - MFC designated the ALCSA chair as a permanent member of its Executive Committee and also its Policy and Fitness committees  - support by mayor (Julián Castro)-> Plan accompanied by policy recommendations, and the mayor’s letter of endorsement  - ALCSA continues to collaborate with both MFC and LHD  - Mayor's Fitness Council (MFC) and LHD routinely request ALCSA input on other local initiatives, including legislative agendas, the county’s Community Health Improvement Plan, and a built environment summit  - integration of ALCSA as a permanent MFC committee to prioritise the adoption and implementation of the plan |  | regulative (coordination), economic  horizontal & vertical | - implementation based on structure and contacts developed during master plan development  - implementation without LHD is only possible because of good network and structures developed during the master plan development - LHD leadership’s late assertion of authority over the plan developed by a volunteer-driven community coalition threatened to undermine the entire effort.  -> appropriate role of LHDs in supporting community coalitions, which are natural advocates for an issue |
| 9  Lee et al., 2012  USA, New York/city/guideline | Active Design Guidelines (ADG)  increasing physical activity in the design and construction of neighbourhoods, streets and buildings | case study  non-reported  not reported | city departments, private associations | Yes  - NYC ADG Team: core city agencies (DOHMH, DDC, City Planning and Transport) -> cooperation with other city agencies  - 3 areas: 1) policy efforts to integrate the ADG into all city building and street construction projects and contract processes and greening of construction codes, 2) outreach to building managers, schools and community groups to encourage elements of the ADG such as the adoption of stair prompts and Playstreet, 3) training of architects and planners (Sep. 2010- Aug. 2011, over1300 architecture, planning, design and real estate professionals in the NYC were trained)  NYC’s Departments of Transportation, City Planning and DDC have been working to increase bicycle infrastructure and further pedestrianised NYC streets by creating pedestrian plazas and streets temporarily closed to cars.  - study underway supported by a second RWJF ALR grant to assess the cost of implementing the ADG in affordable housing in NYC, Atlanta and San Antonio | other departments and professionals | communicative, regulative (coordination)  horizontal & vertical | - cross-sector partnerships - across disciplines and public-private sector partnerships – were necessary since health sectors do not design or build our built environments - a small number of core partners frequently met to get the work done - complementary roles for health partners and built environment partners -> Health partners created forums (Fit City conferences) -> dialogue with design and planning professionals -> contact with design and planning organisations(local chapter of the American Institute of Architects, the American Planning Association and the US Green Building Council) - health partners: information about health priorities and available evidence - Health Department: key role in assisting with and supporting the development and implementation of intersectoral initiatives from ideas generated, including the provision of the city - and grant-funded staff - Research: key role in providing the evidence base for the initiatives and in informing implementation and evaluation |
| 10  Dawson et al., 2015  England, Liverpool/city/strategy | Liverpool Active City Strategy 2005-2010  projects and programmes to promote increased activity among the city’s population | case study  non-reported  interviews (n=13) | health, education, sports and physical activity and transport sectors | Yes  - coordination and branding of existing activities and resources under one Liverpool Active City umbrella -> With encouragement from the Liverpool Active City process with the head of the city council’s United Kingdom Programme Team for review of applications and for making recommendations on funding decisions related to physical activity -> all major projects branded as part of Liverpool Active City  -development of new interventions to expand the programme, add value to existing resources and enhance opportunities for exercise  -> continues to oversee the programme, approves funding for projects and supports the implementation of the city’s physical activity strategy, steers the Liverpool Active City programme and promotes its brand.  - meets quarterly and is co-chaired by the Assistant Director of the city council’s Sports and Recreation Service and the Associate Director of Public Health at Liverpool Primary Care Trust-> Liverpool Active City coordinator reports to the Liverpool Sports and Physical Activity Alliance (SPAA) and takes the lead from the group-> SPAA established an extensive research and evaluation programme through Liverpool John Moores University to assess the progress of Liverpool Active City, inform the various processes and measure their impact - implementation of a neighbourhood focus to ensure that residents in all parts of the city had opportunities to benefit from the programme;  - integration of physical activity elements into broader urban and health agenda -> While there was a longstanding tradition of intersectoral action in Liverpool, notably with the education sector, the physical activity agenda became, over time, integrated strategically with other policy agendas in the city and further developed intersectoral action to achieve common goals  - in 2008, locally-based Active City coordinators were appointed -> facilitated work with communities at the local level, helped to develop local partnerships with a wide array of stakeholders, helped to identify the existence of gaps in provision, and provided the extra capacity to draw in additional funding to local areas  - strategic interaction -> part of the city’s obesity agenda - Links to parks and green spaces  - cooperation with the transport department and engagement of employers and employees from the public, private and voluntary sectors (e.g. Health@Work, commissioned by Liverpool Primary Care Trust) and Football club  - Funding: central government’s Area Based Grant (and the preceding Neighbourhood Renewal Fund), together with money from Sport England and mainstream funding from the Primary Care Trust and city council | members in authorities, civil and community organisations | communicative, regulative (coordination), economic  horizontal and vertical | - those responding to the surveys in Liverpool who were active increasing by 2.5% between 2005-2006 and 2009-2010.   - young people under 16 years of age: proportion has risen -> 2008-2009 and 2009-2010 indicated that the proportion of young people in school years 1-11 who participated in at least three hours of high-quality physical education and out-of-hours school sport increased from 50% to 58%  - 55 000 people now use lifestyle centres within the city - a 43% increase since 2005; - between April 2006 and March 2007, 168 000 people participated in an initiative supported by Active City; - between July 2008 and September 2010, over 7000 individuals participated in at least one physical activity session in the City and North NMA, with one in five joining a fitness centre. - over 1000 new people attend Walk for Health per year; - over 250 new people attend Cycle for Health per year; - 91% of children (in school years 3-6) are now participating in at least 120 minutes of curriculum physical education each week; - Sportslinx organised fun fitness days for 5500 children in 110 primary schools. |
