# Supplementary material for: What we know about the actual implementation process of public physical activity policies: results from a scoping review
Source: Eur J Public Health. 2022 Nov 29;32(Suppl 4):iv59–65. doi: 10.1093/eurpub/ckac089 (PMC9706118; doi:10.1093/eurpub/ckac089)
Supplement: ckac089_Supplementary_Data [file ckac089_supplementary_data.zip › ckac089_Supplementary_Data/Supplementary Appendix4_key terms.docx]

Supplementary Appendix 4

**Used key terms and definitions**

**Public Policy:** a form of government action usually expressed in, e.g., a law, a regulation, guideline, or recommendation and reflecting the intent of the government or its representative entities

**Agenda Setting:** getting the “problem” on the formal policy agenda of issues to be addressed by presidents, cabinet members, parliament, or ministers of health, finance, education, or other relevant ministries

**Formulation:** identify the option, analyse the impact, and define the policy; e.g., legal status, content, wording, structure, responsibilities, resources

**Adoption:** formal adoption of the policy

**Implementation:** responsible agencies (public or private) implement the policy by, e.g., establishing procedures, writing guidance documents, issuing grants, building-up structures, connecting with existing structures

**Monitoring & Evaluation:** short-, medium-, long-term outcome evaluation, process evaluation, impact evaluation as feedback for agenda setting, formulation, adaption, implementation

**Policy instruments**: developed by the government as a way to implement policies and influence the behaviour of citizens and businesses; Classification used here is legal/regulatory, economic (financial), communication (information), and behavioural policy; every instrument has its legal quality

**Implementation strategies:** top-down, bottom-up, mixed (see Table 1 for characteristics)
